# Hemorrhage after Pulp Extirpation

**Published:** 1900-03-15

**Authors:** L. West


Hemorrhage after Pulp Extirpation.—Wind cotton on a broach, dip in 25 percent pyrozone and put up the canal, and the bleeding will stop instantly. The canal can be dried and filled at once.—*L. West, Items of Interest.*

